# CMKb: a web-based prototype for integrating Australian Aboriginal customary medicinal plant knowledge

**DOI:** 10.1186/1471-2105-9-S12-S25

**Published:** 2008-12-12

**Authors:** Jitendra Gaikwad, Varun Khanna, Subramanyam Vemulpad, Joanne Jamie, Jim Kohen, Shoba Ranganathan

**Affiliations:** 1 Department of Chemistry and Biomolecular Sciences, Macquarie University, Sydney, NSW 2109, Australia; 2 Australian Research Council (ARC) Centre of Excellence in Bioinformatics, Macquarie University, Sydney, NSW 2109, Australia; 3 Department of Health and Chiropractic, Macquarie University, Sydney, NSW 2109, Australia; 4 Department of Biological Sciences, Macquarie University, Sydney, NSW 2109, Australia; 5 Department of Biochemistry, Yong Loo Lin School of Medicine, National University of Singapore, 8 Medical Drive, Singapore 117597

## Abstract

**Background:**

The customary medicinal plant knowledge possessed by the Australian Aboriginal people is a significant resource. Published information on it is scattered throughout the literature, in heterogeneous data formats, and is scattered among various Aboriginal communities across Australia, due to a multiplicity of languages. This ancient knowledge is at risk due to loss of biodiversity, cultural impact and the demise of many of its custodians. We have developed the Customary Medicinal Knowledgebase (CMKb), an integrated multidisciplinary resource, to document, conserve and disseminate this knowledge.

**Description:**

CMKb is an online relational database for collating, disseminating, visualising and analysing initially public domain data on customary medicinal plants. The database stores information related to taxonomy, phytochemistry, biogeography, biological activities of customary medicinal plant species as well as images of individual species. The database can be accessed at . Known bioactive molecules are characterized within the chemoinformatics module of CMKb, with functions available for molecular editing and visualization.

**Conclusion:**

CMKb has been developed as a prototype data resource for documenting, integrating, disseminating, analysing multidisciplinary customary medicinal plant data from Australia and to facilitate user-defined complex querying. Each species in CMKb is linked to online resources such as the Integrated Taxonomic Information System (ITIS), NCBI Taxonomy, Australia's SpeciesLinks-Integrated Botanical Information System (IBIS) and Google images. The bioactive compounds are linked to the PubChem database. Overall, CMKb serves as a single knowledgebase for holistic plant-derived therapeutics and can be used as an information resource for biodiversity conservation, to lead discovery and conservation of customary medicinal knowledge.

## Background

Australia is among the 34 biodiversity hotspot countries in the world [1] endowed with unique endemic plant diversity. It is estimated that 85 percent of over 21,000 vascular plant species are endemic to Australia [2]. More than 40,000 years of Aboriginal inhabitation [3] has led to the use of medicinal plants from this vast bioresource for maintaining and treating health-related problems [4]. Aboriginal remedies vary between clans and in different parts of the country, with no single set of aboriginal medicines and remedies [5]. The indigenous knowledge has been passed on from one generation to the next orally through traditional songs, stories, poetry and legends [6]. Unfortunately, Aboriginal customary medicinal knowledge is poorly documented and is on the verge of being lost due to dislocation and the westernisation of the communities [7,8].

Documented Australian medicinal plant knowledge is in the main, fragmented, restricted to specific locales and of limited applicability, usually to pharmacology or phytochemistry. Several studies have focussed on the Northern Territory, where the use of medicinal plants has been documented, with limited data on chemical components and pharmacological assay work [9,10]. A database of plants used as bush foods and medicines by New South Wales Aboriginal communities comprises information largely obtained from published sources or early manuscripts [11], but does not include chemical or pharmacological data. The CSIRO Australian phytochemical database comprises a compendium of published work, searchable by plant and chemical names alone [12]. Thus, there is no single comprehensive inventory of Aboriginal medicinal plants available similar to initiatives such as Native American Ethnobotany database [13] and Prelude Medicinal Plants Database from Africa [14]. The available information in published literature is species-specific, scattered and in different formats, making data integration challenging.

Customary knowledge of medicinal plants and practices is a significant contributor to scientific research and development in pharmaceuticals, cosmetics, foodstuffs, agricultural products and a wide range of other biologically based products and processes [15]. Access to public domain information on Australian customary medicinal plants will advance research in bioinformatics, ethnobotany, taxonomy, biogeography and phytochemistry. Here, we report the development of a comprehensive knowledgebase for Australian customary medicinal plants, CMKb. To the best of our knowledge, this is the first such knowledgebase of its kind.

## Construction and content

### System architecture

**Figure 1:**
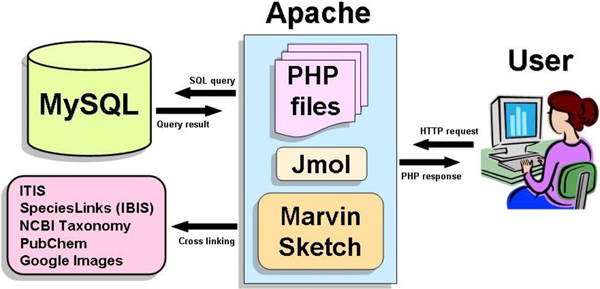
Schematic presentation of system architecture of CMKb.

The goal was to design a database which could be flexible and could accommodate heterogeneous data from published literature or bibliographic search. CMKb is developed using MySQL 5 relational database [16] for systematic and efficient content management. The user-friendly interface, consisting of dynamic web pages, is developed using PHP 5 [17] for data visualisation and data management. The chemoinformatics module incorporates Jmol, a Java based applet program [18] for visualization and Marvin Sketch [19] for drawing and editing of chemical structures. The data is served using Apache webserver [20] (Figure 1).

### Construction method

Before developing the database schema, end user and data resource availability assessment was carried out. The assessment results showed that the potential end users range from members of Aboriginal communities to scientists with interests in ethnobotany, phytochemistry, biology and microbiology. The major data resource is the information collated from an exhaustive literature survey.

**Figure 2:**
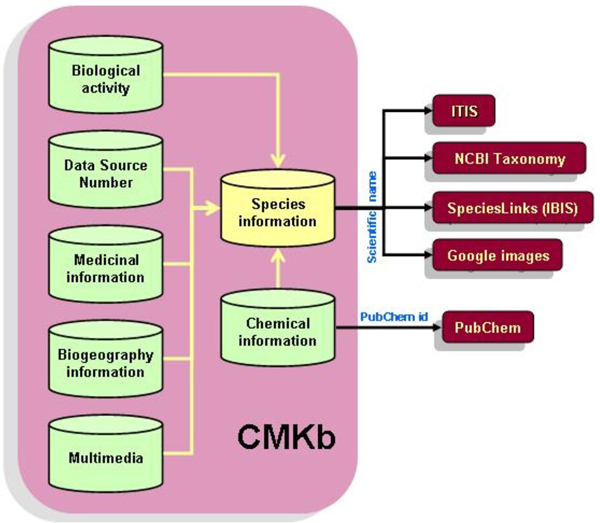
Dataflow in CMKb, showing external links.

We have created a novel schema for integrating multidisciplinary information on medicinal plant species, such as taxonomy, habit and habitat, phytochemistry, bioactivity, biogeography, data sources, medicinal preparation methods and usage, community information, and images into CMKb. Since the species name is the fundamental biological descriptor [21], all the information is linked to the scientific name. Thus, the species information table is central to our schema, and is connected to the other tables (Figure 2). CMKb is designed with the possibility of future expansion including scaling to accommodate very large datasets, and the addition of other multidisciplinary components, described later.

### Content of the database

Information related to medicinal plant species is stored in seven major tables (Figure 2) which are briefly described below. Mandatory information comprises the species name, the published reference and the medicinal use.

#### 1. Species information

Information related to customary medicinal plant species such as kingdom, family, scientific name, synonym, common name, native language name, habit and habitat as well as author citation, is stored in this table. This table is the hub to which all other tables are connected. The scientific name from this table is also used for cross-linking to external data portals, such as IBIS, ITIS and NCBI Taxonomy.

#### 2. Data Source Number (DSN)

Each published article in the literature used to collate and populate the database, is assigned a unique DSN identifier. The DSN table contains fields such as the title of the article, reference type (such as thesis, journal or book), names of authors and citation details.

#### 3. Medicinal information

Species-specific customary medicinal information such as the parts of the plant used, preparation method, taste, odour, colour, application and storage method, is collated in this table.

#### 4. Biological activity information

This table records the biological activity associated with the medicinal plant. The type of assay used to identify biological activity (such as antifungal, antiviral, antibacterial), the specific assay used, assay targets (such as cell line, enzyme and organism name) are recorded in this table.

#### 5. Chemical information

**Figure 3:**
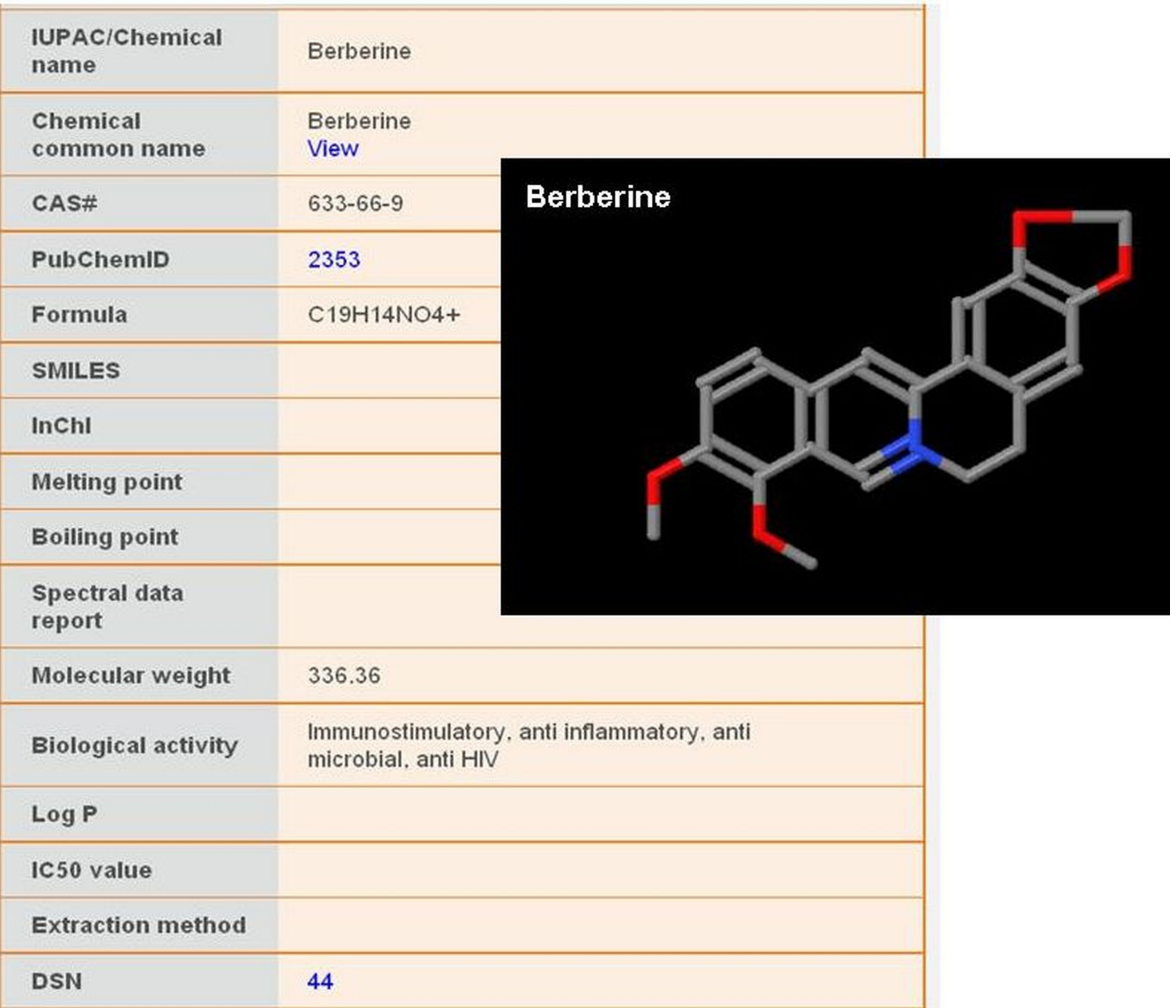
Chemical information page with structure visualization.

This table is used to store the chemical information and structure of bioactives derived from the medicinal plants such as IUPAC name, CAS number, PubChem [20] identifier, common chemical name, chemical structures in SMILES and MOL formats, biological activity related to that chemical compound, spectral data and other physical properties. The chemical structures are created locally using Marvin Sketch and are displayed using Jmol, a freely available Java applet. PubChem identifier stored in this table is used to link to PubChem database [22] from CMKb (Figure 3).

#### 6. Biogeography information

The biogeography table collates observational data of the species from the published literature such as locality name, latitude and longitude in decimal units, district/town, state and country.

#### 7. Multimedia information

CMKb will also accept data in multimedia formats. This table is used to store multimedia information for each species, in the form of videos, drawings and photographs. Multimedia file formats such as jpeg, mpeg and avi can be uploaded to the database, with detailed text description.

## Utility and discussion

CMKb provides a user friendly web interface for accessing and managing the customary medicinal plant data. The database consists of three main modules: ***Browse, Search ***and ***Data Management***. Links to these modules are provided as a menu on the LHS of the CMKb website, as "Browse," "Search" and "Login," respectively. A brief description of each module is given below.

### • Browse module

**Figure 4:**
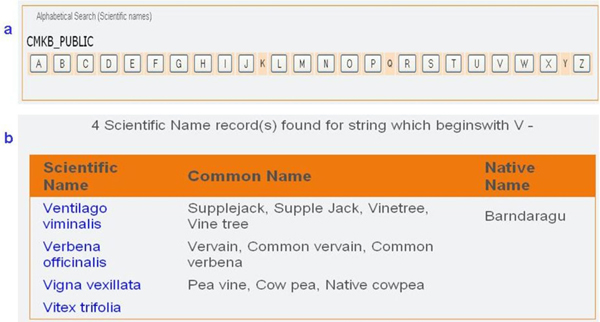
**Browsing the CMKb database**. a. Alphabetical listing of species in the Browse module, and b. a list of species starting with "V".

The database contents can be browsed (Figure 4a) using the alphabetical listing of scientific names and these are hyperlinked to a species list (Figure 4b), each of which is linked to a detailed information page.

Since CMKb is a web-based application, we have provided external links to other relevant global databases and data portals. Linking with other databases providing taxonomic, geospatial and molecular information, and search engines for images will help in data mining and facilitate the exploration of questions that, at present, cannot readily be answered [23] and would provide additional value to the information. Using the scientific name from CMKb we have provided external links to public domain data portals such as Integrated Botanical Information System (IBIS) [24] which provides links to a range of Australian data portals, Integrated Taxonomy Information System (ITIS) [25], NCBI Taxonomy [26,27] and Google images [28] for species images.

### • Search module

**Figure 5:**
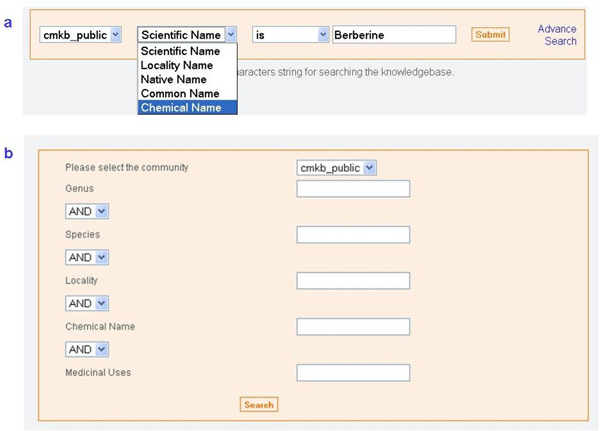
**User-defined querying facilities**. a. Quick search and b. Advanced searching for expert users.

The database can be searched using its comprehensive search engine. The "Quick Search" option provides users with the facility to query the database by scientific name, species common name, native name, locality or chemical name using different logical parameters such as "contains", "begins with", "ends with" and "is" (Figure 5a).

For more complex queries, the "Advanced Search" option can be used, where the user can combine different search fields, using AND as the logical parameter (Figure 5b)

### • Data management module

Efficient online content management is coordinated by CMKb's data management module, accessible to authorized users via the Login link. The data management module is provided with ADD, EDIT and DELETE functionality for managing data present in different tables.

The overall contents of the database can be accessed from the "Content Summary" link.

## Conclusion

Customary Medicinal Knowledgebase (CMKb) is a prototype for collating, integrating, visualising, disseminating and analysing multidisciplinary public domain data on customary medicinal plants. It is a holistic knowledgebase with data on taxonomy, biogeography, ethnobotany, phytochemistry, and bioactivity of the customary medicinal plants used by the Australian Aboriginals. The goal of CMKb is to collate information from scientific publications which are peer reviewed along with documenting and conserving the dwindling customary medicinal plant knowledge. The data will be constantly scrutinised by the experts and will be updated accordingly. Overall, CMKb is developed as a single knowledgebase for holistic plant-derived therapeutic substances and can be used as an integrated resource by researchers, policy makers, students and Aboriginal communities. As the database grows CMKb can be used for research in areas such as Geographical Information System (GIS) studies, chemoinformatics and biodiversity informatics. Further, the goal is to help address global and national priorities of biodiversity conservation, better human health, and smart use of information using information technology.

## Availability and requirements

CMKb is freely available online at 

## List of abbreviations used

**CAS**: Chemical Abstracts Service; **CMKb**: Customary Medicinal Knowledgebase; **CSIRO**: Commonwealth Scientific and Industrial Research Organisation; **DSN**: Data Source Number; **FDSN**: Field Data Source Number; **GIS**: Geographical Information System; **IBIS**: Integrated Botanical Information System; **ITIS**: Integrated Taxonomic Information System; **IUPAC**: International Union of Pure and Applied Chemistry; **JRE**: Java Runtime Environment; **LHS**: Left Hand Side; **NCBI**: National Center for Biotechnology Information; **SMILES**: Simplified Molecular Input Line Entry Specification

## Competing interests

The authors declare that they have no competing interests.

## Authors' contributions

SR, JK, JJ and SV conceived the database concept. JG developed and constructed the database. VK contributed to the web interface and developed the Chemical information module. JG and SR wrote the paper. All authors approved the manuscript and declare that there is no conflict of interest.
